# Organic acid concentration thresholds to mobilize phosphorus (P) in dryland soils

**DOI:** 10.1007/s10533-025-01298-5

**Published:** 2025-12-20

**Authors:** Kalpana Kukreja, Elizabeth Noriega Landa, Wen-Yee Lee, Mark A. Engle, Anthony Darrouzet-Nardi

**Affiliations:** 1 Department of Biological Sciences, University of Texas at El Paso, El Paso, TX USA; 2 Department of Chemistry and Biochemistry, University of Texas at El Paso, El Paso, TX USA; 3 Department of Earth, Environmental and Resource Sciences, University of Texas at El Paso, El Paso, TX USA

**Keywords:** Phosphorus, Organic acids, Soil phosphorus mobilization, Calcium, Dryland critical zone, Jornada Experimental Range

## Abstract

Available phosphorus (P) concentrations are low in dryland soils due to high pH values linked to the presence of pedogenic carbonates. Thus, dissolution and mobilization of P compounds are important controls on P availability for plants, microbes, and biocrusts. One process that has been hypothesized as a way for dryland organisms to acquire P is the exudation of organic acids that can release bound P compounds. To explore this process, we assayed the critical thresholds of organic acid (citrate, malate, and oxalate) concentrations required to mobilize P in a range of dryland soils. Our results showed that: (1) Concentrations of oxalate or citrate, on the order of 1000 µmol/L are needed to effectively mobilize PO_4_^3−^ in all landforms and microsites we examined. (2) The in situ organic acid concentrations in bulk soil core samples were < 100 µmol/L, both under plant canopies and in interspaces, suggesting they are below the needed threshold to mobilize P. However, hot spots such as the rhizosphere, though difficult to quantify, may still be locations where the concentration of organic acids approach the threshold. (3) Oxalate was the most effective in releasing PO_4_^3−^, likely as a result of removing aqueous Ca via calcium oxalate precipitation. Overall, our results show that for P acquisition through organic acid production to be effective in dryland soils, a relatively high threshold of organic acid concentration that substantially exceeds bulk soil concentrations must be reached, suggesting that if it occurs, it is restricted to localized microsites within the soil matrix.

**Supplementary Information:**

The online version contains supplementary material available at 10.1007/s10533-025-01298-5.

## Introduction

In dryland soils, available phosphorus (P) for plants, microbes, and biocrusts is restricted due to several interconnected factors, including the presence of pedogenic carbonates, high pH, and elevated concentrations of elements that bind P, such as Ca and Mg (Lajtha and Schlesinger 1988; Ström et al. 2005; Belnap 2011). As P becomes limited by accumulation in inaccessible pools, solubility conditions that affect P (such as cation concentrations) that determine its presence in soil pore water become increasingly important in making P available. In response to low P availability, plants, microbes, and biocrusts may employ several strategies to access P, including (i) increase of absorptive capacity via increased plant root hair length/density, sometimes via proteoid or cluster roots; (ii) plant increase in C investment in mycorrhizal symbionts to increase exploitation of interspace soil; (iii) secretion of phosphatase enzymes (or upregulation of root membrane phosphatases), which facilitate the hydrolysis of organic P into available P; (iv) plant or microbial acidification of the rhizosphere via the release of H^+^; and (v) plant, biocrust or microbial release of organic acids to act as chelating agents that solubilize inorganic P, thus making it available in soil pore water (Jones 1998; Belnap 2011; Wang et al. 2019; Wang and Lambers 2020; Aslam et al. 2020). In this study, we focus on the relevance of this last and often-hypothesized mechanism in dryland soils, the use of organic acids to chelate P or cations such as Ca^2+^ that bind it, which has the effect of increasing P concentration in soil pore water and making it available to soil organisms (Belnap 2011; Wang and Lambers 2020; de-Bashan et al. 2022).

**Fig. 1:**
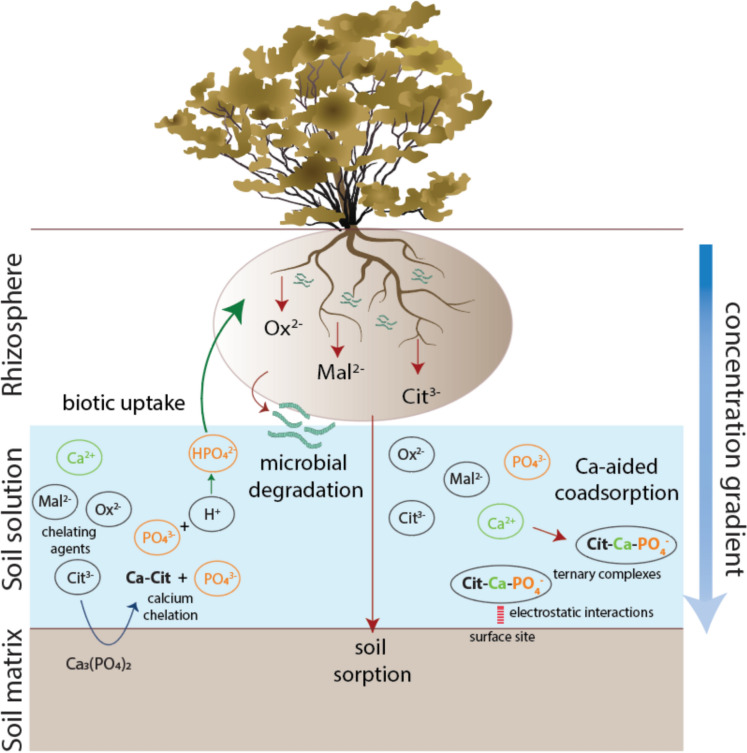
A conceptual figure showing possible interactions of organic acids in dryland soils. The left side of the diagram illustrates how organic acids mediate P acquisition: dryland organisms such as plants and microbes exude organic acids, which are present in the soil in their anionic forms (oxalate, malate, and citrate). The large vertical blue arrow represents the local diffusion gradient of organic acid concentration, which is typically slow (Jones et al. 2003), where dark blue indicates a higher concentration near the rhizosphere, while light blue shows a lower concentration near the soil matrix. To mobilize phosphate, these acids either displace P from the soil matrix or chelate Ca to form complexes (small blue arrow), thereby releasing phosphate into the soil solution and making it readily available for biotic uptake (green arrow). However, there may be other interactions in the soil, such as: 1) microbial degradation, where microbes breakdown these acids (dark red arrow); 2) soil sorption to anion exchange sites (dark red arrow); and 3) Ca-aided co-adsorption, a process that occurs when these acids are present in low to moderate concentrations in the soil solution, leading to the formation of ternary complexes (dark red arrow) between these acids, Ca, and P (citrate-calcium-phosphate). These complexes could either be present in the soil solution (Kubicki et al. 1999) or could bind to the mineral surface through electrostatic interactions (neon red dashed line), which involves outer sphere complexation with no covalent bonding involved (Lackovick et al. 2003; Duputel et al. 2013b; Wang & Lambers 2020). In both cases, P is immobilized, resulting in low concentrations of biotic available P in the soil solution. The colors used for the elements are based on the CPK coloring scheme

Organic acids that are often implicated in mobilization of P in soils include oxalic acid, malic acid, and citric acid (Ström et al. 2002; Menezes-Blackburn et al. 2016; Mora-Macías et al. 2017; Canarini et al. 2019). While in drylands, most organic acids found in soil pore water exist as fully dissociated anions due to the high pH (> 7) (Jones et al. 2003; Wang and Lambers 2020), in this study, we will use the generic term “organic acids” and, wherever required, refer to their anionic forms as citrate, malate, and oxalate. At soil mineral surfaces, these organic acids either compete with P for sorption sites to displace it, or act as chelating agents, binding to form complexes with cations such as Ca^2+^ in alkaline soils and Fe^3+^ and Al^3+^ in acidic soils, thereby releasing P in the form of orthophosphate (H_2_PO_4_^−^ or HPO_4_^2−^), which plants can efficiently uptake (Menezes-Blackburn et al. 2016; Wang et al. 2018; Wang and Lambers 2020; Almeida et al. 2020; de-Bashan et al. 2022) (Fig. 1). In addition to assisting with P acquisition, these acids can also chelate other cations (Fe, K, Mg, Mn, Na) in the soil and help to solubilize micronutrients such as Fe and Mn, which may also be limiting at high pH because of their low solubility (Ström et al. 2005). However, a recent review (Wang and Lambers 2020) has suggested that it may not always be the case that rhizosphere organic acids assist with plant P acquisition or micronutrient chelation in this way. Some factors that may work against this mechanism (Fig. 1) include: (i) the plant, microbe, or biocrust releasing a low amount of organic acids that might not be enough to help mobilize P chemically, with concentrations being below an “effective/critical” concentration (Wang and Lambers 2020). However, these low levels could still help microbes to retain more P in organic form (Menezes-Blackburn et al. 2016); (ii) the organic acids in soil may become sorbed to soil particles or consumed by microorganisms before affecting P availability (Oburger et al. 2011); and (iii) the possibility of ‘Ca-aided co-adsorption’ of organic acids when present in low to moderate concentrations (< 50 µmol/L), a process in which, for example, citrate binds to clay minerals and increase their surface negative charges, thereby enhancing Ca^2+^ ion adsorption, which in turn attracts additional positive charges to the surface sites of minerals, further increasing the PO_4_^3−^ ions’ adsorption through similar electrostatic interactions (Duputel et al. 2013b). These ternary complexes (citrate-calcium-phosphate) could also be present in the soil solution in aqueous form (Kubicki et al. 1999; Lackovic et al. 2003). While it is important in many soils and ecosystems to assess the role of these organic acids in P availability, most studies are limited to agricultural plants grown in pots, rhizobox, or hydroponic culture systems, and represent only a few model ecosystems (Oburger and Jones 2018; Wang and Lambers 2020). Therefore, to gain a better understanding of the role of exuded organic acids in soil P mobilization, further field experiments should be conducted in diverse ecosystems with specific species and soil types (Wang and Lambers 2020).

Beyond P acquisition, the action of plant, biocrust and microbially exuded organic acids in soils are of relevance to chemical weathering, soil formation, and, ultimately, development of the “critical zone,” the canopy-to-groundwater portion of Earth’s surface controlled by biological and geological processes (Drever and Vance 1994; Drever 1994; Richter and Billings 2015; Molina et al. 2019; Finlay et al. 2020; Dawson et al. 2020). However, as with P mobilization, there is a debate in the literature as to the level of impact organic acids have on chemical weathering over longer spatial and temporal scales, mainly due to their low concentrations or rapid turnover rates in the soil solution (Jones 1998; Lawrence et al. 2014). Organic acid concentrations in natural soils are variable and can vary depending on the soil organisms type (plant species, microbes, biocrusts, fungi, etc.), the extent of biological activities taking place, organic matter, and soil type (Jones 1998; Adeleke et al. 2017). To date, several studies have focused on investigating the P extraction efficiency of organic acids through various extraction assays to assess the P availability in the field for different soil types. Among these studies on calcareous soils, it has been observed that the organic acids release P in the following order: oxalate > citrate > malate (Lu et al. 2001; Ström et al. 2002, 2005; Khademi et al. 2009; Zhao and Wu 2014). Furthermore, a study has found that acidic forest soils may have a critical threshold between 10,000–25,000 µmol/L of citric and oxalic acid (Zhu et al. 2022). However, we still lack information on the critical concentration of organic acids that is sufficient to cause a significant increase in soil P availability (Jones et al. 2003) in different soil types in general and particularly in dryland soils, which cover a large portion of Earth’s surface (Prăvălie 2016).

In this study, we explore the relationship between organic acids, P bioavailability, and cation concentrations in dryland soils, with a focus on (i) identifying critical threshold concentrations of various organic acids that can mobilize P and (ii) examining the generality of any such thresholds across dryland soils with varying characteristics such as parent material and pH. Specifically, we hypothesize that, across soil types, dryland plants and microbes use organic acid exudation to release bound P through cation chelation in calcareous soils. While we cannot measure all relevant parameters, such as organic acid production and exudation rates by dryland organisms, we will test one key aspect of the hypothesis: the concentration of organic acids needed to release phosphate; we will also explore the natural concentrations of organic acids found in soils, though with the acknowledged limitation of not being able to characterize concentrations in every relevant microsite, particularly in the rhizosphere. If dryland organisms use organic acid exudation as an effective strategy, we might expect soil organic acid concentrations to approach the thresholds required for P mobilization. To evaluate this, we conducted a laboratory organic acid addition experiment using soil from three sites that varied in desert landforms, as well as two microhabitats (under-plant and interspace). We then quantified phosphate and measured the response of different cations (Ca, K, Mg, Fe, and Mn) to various types and concentrations of organic acids. We also measured the concentration of organic acids in field-collected bulk soils to help constrain the expected field values of in situ organic acid concentrations and help contextualize the critical thresholds for P release that we estimated.

## Methods

### Study site

**Fig. 2:**
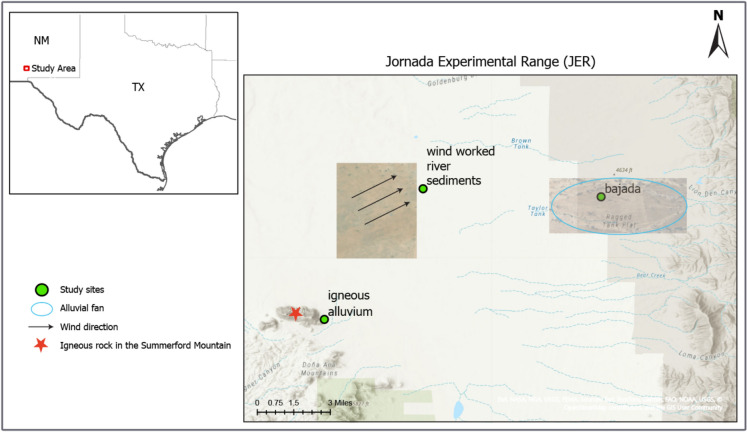
Study map showing site locations of the Jornada Experimental Range

We conducted this study within the Jornada Experimental Range (JER), located outside Las Cruces, New Mexico, in the northern Chihuahuan Desert (32.5829° N, 106.6346° W). Three different sites within JER (Fig. 2) were selected based on landform/parent material and the dominant plant species (Table 1). The three sites were labeled the “bajada” site (a series of coalescing alluvial fans), the “wind-worked river sediments” site, and the “igneous alluvium” site. The climate of the JER is typical of semiarid grasslands, with an annual mean temperature and precipitation of 15.2 °C and 244 mm, respectively, for the period 1991–2020 (Menne and Williams 2012; Hernandez Rosales and Maurer 2022), mainly occurring as localized thunderstorms between July and September. Climatic fluctuations during the Quaternary, topographic positions, and variations in parent material lead to varied soil development within the JER (Gile et al. 1981). The bajada site has Pleistocene alluvium that has developed aridisols with a distinct caliche layer (a semi-continuous calcic/petrocalcic horizons—Stage IV) (Gile et al. 1981). The wind-worked river sediment site is formed by fluvial sediments (0.8–5 Ma in age) deposited by ancestral Rio Grande that are then modified by prevailing southwesterly winds. These also form aridisols. Finally, the igneous alluvium site has sediments that were deposited from adjacent mountain slopes during the Holocene, and form mollisols. The pH at the bajada site was 8.1(Darrouzet-Nardi et al. 2023), the wind-worked river sediment site was 8.18, and the igneous alluvium site was 6.7 (*unpublished data*). The vegetation at the JER is a patchwork of C4 grasslands and C3 shrublands, where shrub encroachment has affected numerous grasslands since grazing began in the late 1800s (Laliberte et al. 2004; Gibbens et al. 2005). The growing season is bimodal, with a green-up and flowering event occurring in April and May, followed by a decline in activity during the hot, dry early summer, and then a second green-up leading to maximum plant growth beginning in mid-July and going until mid-September in association with maximal rainfall in the monsoon season (Jul–Sep) (Havstad et al. 2000).

**Table 1:** Site-specific description

Site	Landform	Parent material	Dominant vegetation
Bajada	Alluvial fan remnants (af)	Alluvium from Sedimentary Bedrock, mainly limestone (l)	shrubland (tarbush – *Flourensia cernua*; mesquite – *Prosopis glandulosa*; creosote – *Larrea tridentata*)
Wind-worked river sediments	Alluvial plain wind worked (ap (w))	Sediments derived from Ancestral Rio Grande (aRG)	grassland (black grama – *Bouteloua eriopoda*); biocrust (light cyanobacterial biocrust – *Microcoleus vaginatus*)
Igneous alluvium	Alluvial fan collar and pediments (afc_ped)	Igneous Bedrock (m)	mixed grassland (native black grama grass – *Bouteloua eriopoda*; introduced Lehmann lovegrass – *Eragrostis lehmanniana*)

To test our hypothesis, we first collected soil core samples from the field and then conducted a laboratory experiment. This involved soil extractions using different concentrations and types of organic acids to determine the threshold for P mobilization. The filtrate from these extractions was then characterized for water-soluble ion chemistry. Next, we measured the in situ concentrations of organic acids in these soils. Finally, we compared the in situ organic acid concentrations to the threshold needed to release P.

### Soil collection and organic acid-addition experiment

Soil cores were collected in September 2022 (about two weeks after a 16.5 mm rain event) at a depth of 0–10 cm (core diameter = 5 cm) at each site. Cores were taken under the canopy of 5 representative individuals of the dominant plant species at each site and a nearby interspace sample within each landform/parent material (*n* = 5 each for canopy and interspace). Soil cores were sieved (< 2 mm), removing roots and visible organic matter and homogenizing it before subsampling for extractions. Sub-samples (3 sites × 2 microhabitats × 1 depth × 5 replicate cores × 7 extraction concentrations × 3 organic acids = 630 assays of 30 soil total samples) were extracted at a ratio of 1:5 (m/v) using deionized water (control) as one treatment, and six other treatments were also applied with varying concentrations of three organic acids (oxalic acid, malic acid, citric acid; Table 2). The organic acids solutions were prepared using their sodium salt forms. 3 g of soil were extracted into 15 ml solutions using 50 ml centrifuge tubes, which were shaken for 30 min on a rocking platform, followed by centrifugation (Thermo Scientific Legend XTR) at 4100 rpm for 10 min at 4 °C. The supernatant was then filtered through 0.2 µm cellulose acetate membrane filters (Sartorius). Phosphate concentrations of filtrate were determined using the malachite green colorimetric assay (D’Angelo et al. 2001).

**Table 2:** Description of treatments

Treatment	Organic acids (oxalic acid, malic acid, citric acid) concentration (µmol/L)
T1 (Control, DI water)	0
T2	10
T3	20
T4	50
T5	100
T6	1,000
T7	10,000

The selected concentration range (10 µmol/L–10,000 µmol/L) covers the values commonly measured in soil (1–100 µmol/L), at the root surface (< 1000 µmol/L) except in some plants with cluster roots that could exceed 50,000 µmol/L (Jones et al. 1996, 2003; Jones 1998; Strobel 2001; Ström et al. 2002; Oburger et al. 2011). The highest concentration of 10,000 µmol/L was selected to match and compare with the previous studies, where this concentration was used to evaluate organic acid extraction efficiency (Ström et al. 2005; Khademi et al. 2009; Oburger et al. 2011), to determine the threshold concentration (Menezes-Blackburn et al. 2016; Yang et al. 2019; Zhu et al. 2022), and to release the citrate-extractable phosphorus pool (Crain et al. 2018). Briefly, this citrate extraction (which quantifies the P pool sorbed to compounds of Ca, Al, or Fe, emulating organic acid release by plants and microbes) is one of the four extraction methods based on an increasingly used approach to evaluate biologically based phosphorus pools in soils (DeLuca et al. 2015). Also, we selected these specific organic acids due to their prevalent natural occurrence and frequent use in studies based on measuring extraction efficiency for mobilizing inorganic P in the soils (Ström et al. 2002; Jones et al. 2003; Vance 2008; Belnap 2011; Mora-Macías et al. 2017; Canarini et al. 2019).

Finally, to reduce the possibility of microbial breakdown of the organic acids during the procedure, we 1) extracted them at a low temperature in a walk-in refrigerator at 4 °C, shown to result in higher P yields than extractions performed at room temperature (20 °C) (Magid and Nielsen 1992); 2) used a specific 0.2 µm cellulose acetate membrane filter; and 3) performed a rapid extraction with respect to the half-life of the organic acids (oxalic acid, citric acid = 0.5–12 h; malic = 1–40 min or up to 3 h) reported in the literature (Ström et al. 2001; Adeleke et al. 2017).

### Ion chemistry and associated analysis

Using filtered extracts, the total dissolved element concentrations (Ca, Fe, K, Mg, Mn, Na, and P) were determined by inductively coupled plasma–optical emission spectroscopy (ICP-OES, Horiba Scientific). We excluded the data for Na concentrations in the results as we used organic acids in their sodium forms during soil extractions. Details of the method comparison (malachite green colorimetric assay vs. ICP-OES) for P analysis are available in Supplementary Information (Appendix 1). Additionally, as per Hou et al. (2018), we also investigated the possibility of an organic P pool that may interact with organic acids. In that study, the difference between total dissolved P (ICP-OES) and inorganic P (malachite green colorimetric assay) was considered as organic P. The organic P fraction is an estimated measure, and may include suspended colloidal P, soluble Ca-P complexes, and polyphosphates (Shwiekh et al. 2013; Hou et al. 2018).

In addition to these water extracts, we also determined the total elemental concentrations of these same elements in bulk soil using hydraulic press/x-ray fluorescence (XRF, a composite sample from each site was made into a pellet using hydraulic press followed by XRF). For the lighter element carbon, soils were packed in Sn capsules and measured by elemental analyzer (ECS 4010, Costech Analytical, Valencia, CA) at the Stable Isotope Core Laboratory, Washington State University. Soil inorganic carbon was measured using a calorimeter-based technique (Fonnesbeck et al. 2013). The difference between the total carbon and soil inorganic carbon is reported as soil organic carbon.

### Quantification of in situ organic acid concentrations

Collecting rhizosphere soil was challenging because the sandy soils at our sites do not adhere well to the roots, especially in these water-limited ecosystems. Therefore, we measured bulk soil, since it was feasible to extract and analyze in these dryland soils. We also took an additional step to compare microhabitats (under plant canopies vs. interspace—away from plant canopies). The acid concentrations were estimated in rough orders of magnitude for the production rate based on the published half-times of these acids (Ström et al. 2001; Duputel et al. 2013b; Adeleke et al. 2017). Soil preparation and metabolite extraction steps were performed using the protocol from Swenson and Northen (2019). The pre-and post-drying soil weight was measured to determine the percentage of water weight in the original sample. Each soil sample (*n* = 5 for each site) was placed in a 50-ml polypropylene tube for lyophilization (an alternative to heating to minimize metabolite breakdown) and, subsequently, frozen at − 80 °C. Samples were placed in a precooled lyophilizer (temperature: − 80 °C and vacuum: 0.017 mbar; Labconco) after covering the top of each tube with a single layer of Kimwipe to prevent cross-contamination and were dried (~ 24 h) completely. Dried soils were sieved using a 2 mm mesh in a collection pan and were sterilized in between samples by spraying with 70% ethanol and wiping it clean. For metabolite extraction, in each (2 g) soil sample, 8 mL of LC–MS grade water was added. The sample was placed on a refrigerated orbital shaker at 200 rpm for 1 h, followed by centrifugation at 3220 ×*g* for 15 min (at 4 °C). The supernatants were decanted into a luer-lock syringe (10 mL) fitted to a 0.45 μm (32 mm) filter disc (Pall Acrodisc Supor membrane) and filtered into 15 mL polypropylene tubes. The extracts were frozen immediately at − 80 °C and lyophilized for 6–8 h or until dry.

To determine the organic acid concentrations in the filtered supernatants of the samples, we prepared a standard solution in methanol with organic acid concentrations ranging from 0–300 mg/L, equivalent to 0–2540 µmol/L for oxalic acid, 0–1850 µmol/L for malic acid, and 0–1282 µmol/L for citric acid. To set up the calibration curve for each organic acid, we simultaneously analyzed all peak area values (not the average) with a gas chromatography-mass spectrometry (GC–MS) system and plotted them as a function of the concentrations of the organic acid. To enhance the response of the organic acids in GC–MS, derivatization is necessary to increase their non-polarity. Dried samples were resuspended in 300 µL of methanol, followed by derivatization adapted according to the method (Encerrado Manriquez 2020). We added a mixture of CH_3_OH: HCl: CHCl_3_ (10:3:1) to the sample and sonicated for 1 h at 60–70 °C. The organic acid concentrations were determined according to the parameters reported in the Supplementary Information (Appendix 2). Briefly, 3 µL of the sample were analyzed in an 8890/5977B GCMS (Agilent Technologies, Wilmington DE) coupled with a cold injection system (CIS4, Gerstel) and a thermal desorption unit (TD 3.5 + , Gerstel). The quantification of organic acids was performed using the calibration curve, and the qualitative analysis was performed using the National Institute of Standards and Technology Library (NIST17) library on Agilent’s MassHunter software. Our method did not work for quantifying malic acid.

### Comparison of in situ and threshold concentrations of organic acids

To make quantitative comparisons between the organic acid concentrations used for soil extractions and the organic acid concentrations measured via GC–MS, we estimated the in situ concentrations with the assumption of saturated soil. To calculate the amount of water in saturated soil, we used bulk density and particle density to estimate porosity. For calculations, we used the particle density of calcite (2.71 g cm^−3^) for the bajada and the wind-worked river sediment site and the particle density of quartz (2.65 g cm^−3^) for igneous alluvium site based on the known mineralogy, and the bulk density at each site as bajada—1.46 g cm^−3^; wind-worked river sediments—1.60 g cm^−3^ (Gile and Grossman 1979); and igneous alluvium—1.50 g cm^−3^.

### Data analysis

A linear mixed-effects model was utilized for the analysis (Pinheiro and Bates 2000) using the “nlme” package in R 4.4.0 (R Core Team 2024) with landform/parent material, microhabitat, and treatments as fixed effects and soil cores as random effects since each core was subsampled for each treatment. We then performed a Type 1 ANOVA to determine the significance of the fixed effects and their interactions. Additionally, for significant effects, we used the “emmeans” package to examine specific contrasts using either the Dunnett test to compare organic acid treatments with the control or the Tukey test for pairwise comparisons of site differences (*p* < 0.05) (Hothorn et al. 2008).

## Results

### Threshold concentration of organic acids for P mobilization

**Fig. 3:**
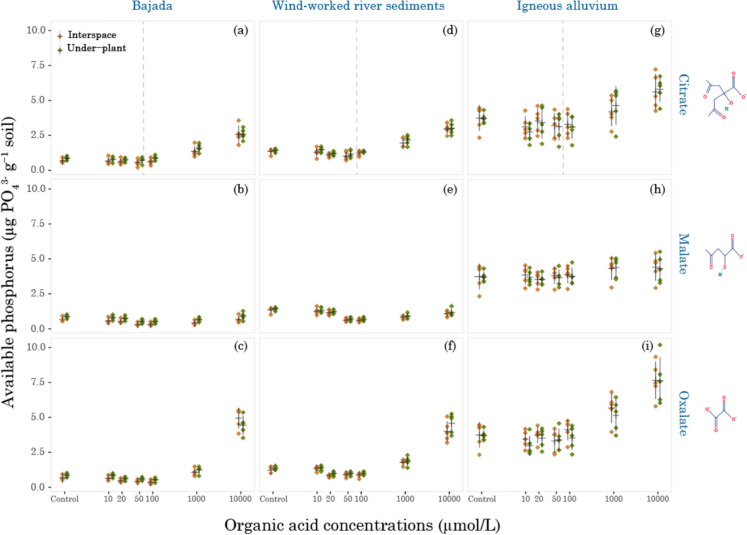
Available phosphorus as a response to varying organic acids, concentrations, landforms, and microhabitats. The X-axis is logarithmic, and each PO_4_^3−^ value (mean ± SE; denoted by a black cross) is the average of five sampling locations at each site. The grey dotted line in the citrate panel denotes the actual concentration (average of the microhabitats) measured in the soil (Refer to Table 4)

In our soils, mobilization of PO_4_^3−^ was only observed at higher concentrations of organic acids (1000 µmol/L and 10,000 µmol/L). This range was consistent for citrate and oxalate, but not for malate, across the landforms, parent material, and microhabitats (Fig. 3). Overall, the fixed effect of organic acid treatments was significant (*p* < 0.0001, *F* = 483.6, Table A3-1, panel A). All interaction effects were tested; only a few (treatment × organic acid and treatment × organic acid × landform) were significant, while others, including those with microhabitat, were negligible. The detailed results of the ANOVA are provided in Supplementary Information (Appendix 3, Table A3-1, panel A). The specific contrast of treatments within each organic acid (Appendix 3, Table A3-2, panels A-C) indicated a continuous decrease in PO_4_^3−^ levels at lower concentrations (10 µmol/L–100 µmol/L) compared to the control, with significant differences at 50 µmol/L (*p* =  < 0.001) for all tested organic acids and at 100 µmol/L for malate (*p* = 0.0006) and oxalate (*p* = 0.03); citrate showed a trend toward significance (*p* = 0.051). At an organic acid concentration of 1000 µmol/L (p < 0.0001), citrate and oxalate increased PO_4_^3−^ concentrations in dry soil by 0.68 µg g^−1^ (35%) and 0.84 µg g^−1^ (43%), respectively. At a high concentration of 10,000 µmol/L (*p* < 0.0001), citrate and oxalate released 1.79 µg g^−1^ (106%) and 3.57 µg g^−1^ (182%), respectively. No significant difference was observed in PO_4_^3−^ concentrations with malate. In contrast, organic P concentrations at 10,000 µmol/L were higher only with malate (*p* = 0.0002) at the wind-worked river sediment site, and with oxalate (*p* < 0.0001) at the igneous alluvium site.

At the higher concentrations in which P mobilization was observed, the extraction efficiencies were oxalate > citrate > malate. The differences were significant (*p* < 0.0001, *F* = 167.83, Table A3-1, panel A). The highest P levels at 10,000 µmol/L concentration were found at the igneous alluvium site (Fig. 3), where PO_4_^3−^ released by oxalate was 7.65 ± 0.59 µg g^−1^ dry soil (Fig. 3 (i), interspace), citrate was 5.79 ± 0.39 µg g^−1^ dry soil (Fig. 3 (g), under-plant) and malate was 4.40 ± 0.43 µg g^−1^ dry soil (Fig. 3h, interspace).

PO_4_^3−^ availability varied significantly across the studied sites with varying landforms and parent material (*p* < 0.0001, *F* = 142.19, Table A3-1, panel A). The phosphate levels were highest at the igneous alluvium site, followed by the wind-worked river sediments site and the bajada site. The mean phosphate level at the igneous alluvium site was 4.03 µg g^−1^ dry soil (3.74, 4.31; 95% CI), which was 2.60 µg g^−1^ higher than the mean phosphate level at the wind-worked river sediments site, which was 1.43 µg g^−1^ (1.15, 1.72; 95% CI; *p* < 0.0001). While the phosphate level at the igneous alluvium site was 3.04 µg g^−1^ higher than that of the bajada site, with a mean of 0.98 µg g^−1^ (0.70, 1.27; 95% CI; *p* < 0.0001). These results matched the pattern of the total P levels (as measured by hydraulic press/XRF) at the igneous alluvium site (0.11%; Table 3), which were followed by the bajada site (0.07%) and the wind-worked river sediments site (0.05%).

**Table 3:** (a) XRF elemental analysis (%) and (b) Total carbon, soil inorganic carbon (SIC), and soil organic carbon (SOC) %

Site	Total Ca	Total Fe	Total K	Total Mg	Total P	Total Mn	Total C	SIC	SOC
Bajada	5.94	1.93	1.70	1.11	0.07	0.02	1.73	1.07	0.65
Wind-worked river sediments	1.46	1.48	1.99	0.96	0.05	0.01	0.36	0.18	0.17
Igneous alluvium	1.55	2.88	2.05	1.52	0.11	0.05	0.27	0.03	0.24

The value represents the mean for each site (for reference, 5.94% = 59,400 ppm)

Inorganic P concentration differences between plant canopies and interspace microhabitats were not significant (*p* = 0.72, *F* = 0.13, Table A3-1, panel A), and this trend was consistent across landforms, organic acids, and treatments. In contrast, a trend towards significance (*p* = 0.058) was observed, where organic P levels were higher under plant canopies than in the interspaces (Supplementary Appendix 4, Figure A4-1). In some cases, we detected negative organic *P* values (where inorganic P exceeds total dissolved P), possibly due to analytical uncertainties such as calibration differences, matrix effects, or concentrations below detection limits.

### Cation responses to variable organic acid concentrations

**Fig. 4:**
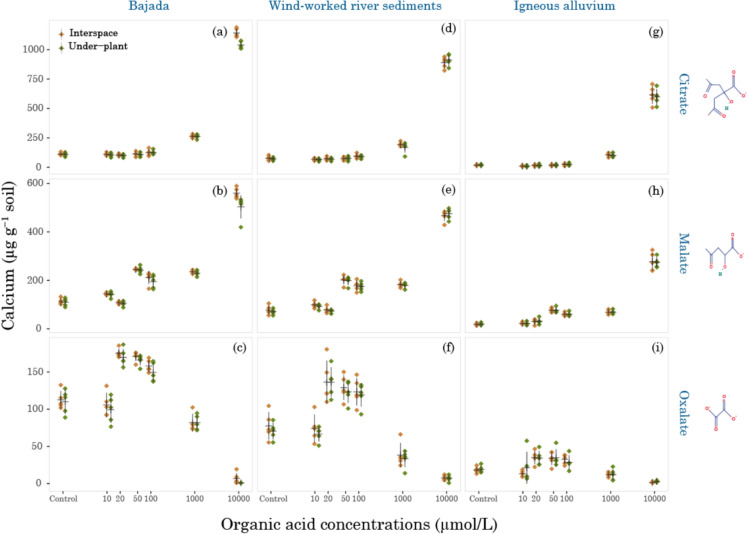
Extractable calcium trends in different organic acids, landforms, and microhabitats. The X-axis is logarithmic, and each Ca^2+^ value (mean ± SE; denoted by a black cross) is the average of five sampling locations at each site

In our tested soils, the behavior of Ca^2+^ ions in the presence of citrate, malate, and oxalate showed several clear trends (Fig. 4). The treatment differences across the studied sites were significant (*p* < 0.0001, *F* = 6300.13, Table A3-1, panel B). The interactions were tested, and the ANOVA results are provided in Supplementary Information (Appendix 3, Table A3-1, panel B). Only a few (treatment × organic acid, treatment × organic acid × landform, and treatment × microhabitat × landform) were significant, while others, including those with microhabitat, were non-significant. Specific contrast of treatments within each organic acid (Appendix 3, Table A3-3, panel A) showed that citrate released calcium only at the higher concentrations of 1000 µmol/L (*p* < 0.0001) and 10,000 µmol/L (*p* < 0.0001), which represented increase of 169% and 1174% respectively, compared to the control. While malate (Appendix 3, Table A3-3, panel B) caused a slight fluctuation in calcium concentration between 10 µmol/L to 100 µmol/L concentration, which was significant at 50 µmol/L (*p* < 0.0001) and 100 µmol/L (*p* < 0.0001). Malate does increase the calcium concentration by 137% at 1000 (*p* < 0.0001) and by 526% at 10,000 µmol/L (*p* < 0.0001). The pattern of calcium released by oxalate (Appendix 3, Table A3-3, panel C) was different from that of citrate and malate. When compared to the control, at 10 µmol/L, there was a slight decrease, and then the calcium concentration increased in the middle concentration range of 20 µmol/L to 100 µmol/L (*p* < 0.0001). However, the pattern changed in the higher concentration of oxalate, and there was a decrease of 37% at 1000 (*p* < 0.0001) and 93% at 10,000 µmol/L (*p* < 0.0001).

Calcium extraction efficiencies were citrate > malate > oxalate. The differences were significant among the organic acids examined (*p* < 0.0001, *F* = 3721.43, Table A3-1, panel B). At 10,000 µmol/L, the highest Ca^2+^ concentration was found at the bajada site, where citrate extracted 1141 ± 17 µg g^−1^ dry soil (Fig. 4a, interspace), malate extracted 560 ± 9.64 µg g^−1^ dry soil (Fig. 4b, interspace), and while oxalate only extracted 6.84 ± 3.43 µg g^−1^ dry soil (Fig. 4c, interspace).

Calcium concentrations significantly differed across the studied sites (*p* < 0.0001, *F* = 369.94, Table A3-1, panel B). Higher Ca^2+^ concentrations were found at the bajada site (204 [196, 211]; mean [95% CI]), which was 46 µg g^−1^ (*p* = 0.004) higher than at the wind-worked river sediment site (157 [150, 165]; mean [95% CI]) and 132 µg g^−1^ (*p* < 0.0001) higher than at the igneous alluvium site (71.3 [64.1, 78.5]; mean [95% CI]). Additionally, the total Ca concentrations measured by hydraulic press/XRF (Table 3) were also highest at the bajada site (5.9% of soil mass), consistent with the trend in the dissolved cations, followed by the igneous alluvium site (1.5% of soil mass) and wind-worked river sediment site (1.4% of soil mass).

The microhabitat exhibited similar results across different treatments, organic acids, and landforms (*p* = 0.20, *F* = 1.72, Table A3-1, panel B).

**Fig. 5:**
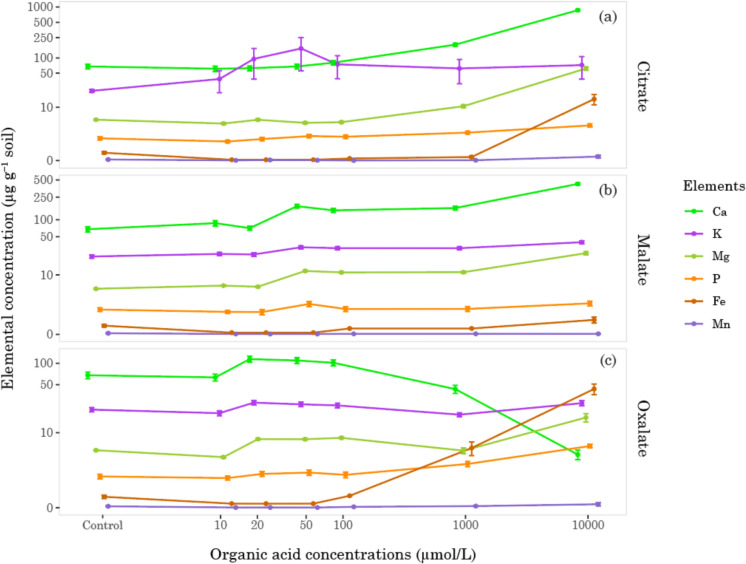
Trend of examined elements in different organic acids concentrations. The X-axis and Y-axis are logarithmic, and symbols represent mean ± SE. The color assigned to each element represents the CPK-coloring scheme

Among other measured cations, for K and Mg concentrations, the organic acid extraction efficiency at 10,000 µmol/L was citrate > malate > oxalate. Meanwhile, for Fe, it was oxalate > citrate > malate, and for Mn, with a minor difference, it was citrate > oxalate > malate (Fig. 5). The results from hydraulic press/XRF measurements showed that, except for total Ca, all other total cation concentrations (Fe, K, Mg, Mn) were highest at the igneous alluvium site, followed by the bajada and the wind-worked river sediments site (Table 3).

### Bulk soil concentrations of organic acids

The site mean for the concentration of citric acids (Table 4) at the studied sites was consistent with the reference range in soil solutions (below 100 µmol/L kg^−1^ soil). We did not find any significant differences between the microhabitats. The oxalic acid concentrations were below the limit of quantification (LOQ).

**Table 4:** Results of GC–MS analysis

Site	Microhabitat	Oxalic acid	Citric acid (µmol kg^−1^ soil)	Citric acid (µmol/L)^b^
Bajada	Under plant	< LOQ^a^	20.8 ± 0.61	65.9
	Interspace	< LOQ^a^	20.4 ± 0.25	64.6
Wind-worked river sediments	Under plant	< LOQ^a^	21.8 ± 0.39	85
	Interspace	< LOQ^a^	21.4 ± 0.71	83.3
Igneous alluvium	Under plant	< LOQ^a^	23.3 ± 1.56	81.3
	Interspace	< LOQ^a^	22.6 ± 0.89	78.7

Values represent each site’s mean ± SE (n = 5)

a—LOQ =  < 4 ppm. b—Estimated for saturated soil. Please refer to the comparison of in situ and threshold concentrations of organic acids section for calculations

## Discussion

In this study, we explored the association between organic acids and P bioavailability by estimating P release thresholds and in situ concentrations in dryland soils of varying parent materials and characteristics using oxalate, malate, and citrate in the 10–10,000 µmol/L concentration range. We found a consistent threshold on the order of 1000 µmol/L for increased P availability across different organic acids (citrate and oxalate) and across soil types from different landforms within the Jornada Experimental Range. These consistencies suggest that this threshold may be a general geochemical feature of calcareous dryland soils, at least in areas with similar climate, rather than varying substantially within a landscape. Additional discussion on site differences is provided in the Supplementary Information (Appendix 5). The high PO_4_^3−^ levels we observed (Fig. 3) at organic acid concentrations of 1000 µmol/L and 10,000 µmol/L were consistent with the previous studies in showing the chelation effects of these compounds in the soil matrix (Ström et al. 2002; Khademi et al. 2009). In the soils we examined, the extraction efficiency of organic acids in releasing phosphorus (oxalate > citrate > malate) and the dominating cation calcium (citrate > malate > oxalate) was consistent with the previous studies in soils from other biomes (Ström et al. 2005; Khademi et al. 2009; Wang et al. 2015; Nezami and Malakouti 2016). Thus, if the concentrations in these desert soils exceed the threshold, our results suggest that oxalate may be most effective in releasing phosphorus. This release likely occurs through interaction with the dominant cation, calcium, either by chelation or competition, thereby replacing PO_4_^3−^, and increasing its concentration in the soil solution.

Our measurements of the in situ concentrations of organic acids in bulk soils revealed that the measured concentrations were low (< 100 µmol/L; Table 4), consistent with previous findings (Hue et al. 1986; Ström et al. 2001; Strobel 2001; Jones et al. 2003; Oburger et al. 2011; Adeleke et al. 2017). Specifically, oxalate concentrations were below the limit of quantification (LOQ), indicating it was present only in trace quantities. This is consistent with previous findings suggesting that oxalate concentrations in soil solutions, both in the rhizosphere and bulk soils, typically do not surpass 50 µmol/L due to their tendency to form salts with low solubility, which eventually results in calcium oxalate formation (Shen et al. 1996; Ström 1997; Ström et al. 2001; Jones et al. 2003). Similarly, low citrate concentrations observed in soil solutions in our study may have resulted from the susceptibility of citrate and malate to rapid microbial breakdown, contributing to their low or below-detection levels (Ström et al. 2001; Mimmo et al. 2008).

Although we initially predicted that we would see clear differences between microhabitats (under plant canopies vs. interspaces—away from plant canopies), no significant differences in either threshold values or in situ concentrations of organic acids were observed. A possible reason for this lack of difference is that our under vs. away sampling scheme may not be the best proxy for exploring the influence of the rhizosphere, with our lack of ability to get soil directly adjacent to roots being an issue. Another possibility is that plant roots efficiently explore the whole soil volume, so being right under the plant does not necessarily mean root density is far greater there. Additionally, while 2022 was a wet year, with around 55% (approximately 132 mm) of the average annual precipitation falling between July and September (Anderson 2024), we suspect that prior moisture conditions would not change the underlying geochemical processes, such as calcium binding of phosphate or chelation. However, an indirect effect on the biotic activity, such as plant growth, and enhanced microbial activity (Hartley et al. 2007; Belnap 2011; de-Bashan et al. 2022) may have influenced the distribution of P in soils across different microhabitats, leading to more consistent P levels. In contrast, organic P was slightly higher in the under-plant microsites than in the interspaces (Supplementary Appendix 4, Figure A4-1), likely due to more organic matter and increased microbial activity in the under plant canopies (Belnap 2011).

A previous study at our same wind-worked sediment site suggested that organic acid exudation might be a viable strategy to acquire P (Crain et al. 2018). The results of the current study suggest that for this to be true, a critical concentration on the order of 1000 µmol/L is needed to solubilize additional P (Fig. 3). This higher concentration is seemingly at odds with the much lower organic acid concentrations we measured in the bulk soils at these sites as described above (< 100 µmol/L). However, it is still possible that in some microsites, such as the rhizosphere, this critical concentration is approached or exceeded, given that localized or temporally present hot spots of high organic acid concentrations could still exist (Oburger et al. 2011). The rhizosphere, in particular, would be a likely location to contain these higher organic acid concentrations, with some studies showing that at the root surface, there may be around ten times higher concentrations than those present in soil solutions, which could put these concentrations close to the 1000 µmol/L (1 mmol/L) threshold. Some studies have even suggested plants with proteoid roots could create concentrations of 50,000 µmol/L (Jones et al. 1996; Strobel 2001; Oburger et al. 2011). However, these hotspots are highly concentrated and their size may be only a few microns in width due to the slow diffusion of these acids in most soils (Jones et al. 2003). While we were not able to document anything like these higher concentrations in our soils, or even concentration differences between under and away from plant canopies, it is an area that clearly warrants further research. Approaches such as leaching bare roots, quantifying exudation rates in the lab or in situ (Phillips et al. 2008) could help better estimate rhizosphere concentrations. Additionally, combining isotope-labeled organic acids with nanoscale approaches such as NanoSIMS (nanoscale secondary ion mass spectrometry) to locate C exudation hotspots on a nanometer scale, or µXANES (X-ray absorption near edge structure) to analyze P speciation at the microscale (Vidal et al. 2021; Wen et al. 2022; Schweizer et al. 2023), along with rhizosphere concentration measurements, could be valuable to provide an overall understanding of P acquisition in drylands.

P demand by plants and microbes is also an important factor to consider. Even in our water extraction controls, soluble P concentrations were not zero, suggesting some baseline availability. In deserts, where productivity is low, plants may not have copious carbon to allocate toward root-exuded organic acids. Thus, depending on the P demand of desert soil organisms, we can speculate that even if the P is occluded by calcium, there might be enough available P in the soil solution, which is sufficient for plants, microbes, and biocrusts, given their relatively low growth rates in this water-limited ecosystem. Though timing of this P demand could be a factor: during the peak growing season (July–September), when P demand is highest, organisms may rely more heavily on additional P inputs through mechanisms such as the recycling of organic P via phosphatases (Rosling et al. 2016). Low concentrations of organic acids could also play an indirect role. Instead of serving primarily as a direct solubilization mechanism, they may stimulate microbial activity more effectively than glucose (Zhu et al. 2022), leading to microbial biomass P accumulation (Menezes-Blackburn et al. 2016) and potentially promoting phosphatase production that can access organic P through enzyme hydrolysis. If plants directly invest in producing these enzymes, they might need additional nitrogen, which could also be limited in these drylands (Belnap 2011). Evidence from subtropical forests shows that organic acids can mobilize 2.5 times more organic P than inorganic P (Hou et al. 2018); in contrast, our own data indicate that organic P represents only around 25% as much as that of inorganic P (Supplementary Appendix 4, Figure A4-1). Even so, using a low concentration of organic acid could still be a viable option to release P via organic P in these dryland soils.

Our water-extractable Ca^2+^ data also help to contextualize the geochemical interactions we observed. Considering that P is thought to be predominantly bound to Ca-P complexes in dryland soils, the trends of Ca^2+^ in the presence of these three organic acids are relevant. Ca^2+^ concentrations increased in the presence of citrate and malate, particularly at higher concentrations. In particular, the calcium-oxalate interaction was worth noting in the presence of oxalate. In contrast to the PO_4_^3−^ levels, Ca^2+^ concentrations increased in the mid-acid concentration range of 20–100 µmol/L, suggesting the formation of soluble calcium-oxalate complexes in solution (Uren 2018), which increases the free calcium ion concentration. However, this trend changes at higher concentrations of 1000 µmol/L and 10,000 µmol/L, as oxalate tends to form calcium-oxalate precipitates. The ability of oxalate to chelate Ca^2+^ could be suggested by a decrease in soluble cation concentrations in soil solution (Wang et al. 2015), which was clearly demonstrated in our study. The trend of decreasing Ca^2+^ levels at higher oxalate concentrations was similar to findings in previous studies on calcareous soils from other biomes (Ström et al. 2005; Khademi et al. 2009). In contrast to citrate and malate, oxalate behavior could be explained by its (i) strong ability to chelate calcium (Wang et al. 2015), (ii) displacement of P through rapid sorption to the soil matrix, and (iii) resistance to microbial degradation (Ström et al. 2001). All these factors contribute to the formation of calcium oxalate and its subsequent precipitation, which favors P dissolution and thus increases P bioavailability.

The total solid-phase elemental concentrations in bulk soils measured with XRF/hydraulic press showed that among the elements of interest for our study, Ca was the dominant cation-forming element at the bajada site, K at the wind-worked river sediment site, and Fe at the igneous alluvium site (Table 3). These elemental profiles may be relevant to P because, for example, as organic acid concentrations get higher, they also interact with other elements, such as Fe, which can potentially make P less available. Our Fe data suggest that these organic acids, particularly oxalate, help solubilize Fe in the tested soils. When oxalate binds with calcium, it might release some Fe, limiting P availability. This is particularly true for the igneous alluvium site, which is slightly more acidic compared to the other sites. The Fe extraction efficiency was similar to a previous study (Ström et al. 2005), but in that study, the effect was observed at 50,000 µmol/L, whereas we saw it at 10,000 µmol/L. Meanwhile, Mg extraction efficiency followed a similar trend as Ca, where we observed an increase of around 12 times in Mg concentrations with 10,000 µmol/L added citrate. In contrast, there was no significant difference in the response of Mn levels to any of the tested organic acids and their various concentrations. These results are consistent with a previous study that found no Mn mobilization with 500 µmol/L of added citrate and malate across all seven tested soils (Jones and Darrah 1994).

Finally, it is interesting to note that our PO_4_^3−^ data suggested that at the lower concentration range of organic acids (10–100 µmol/L), there is a small *decline* in P levels compared to the control, which was most pronounced at 50 µmol/L (Supplementary Appendix 3, Table A3-2, Panels A-C). Similarly, a study conducted on a chromic Cambisol in a Mediterranean shrubland ecosystem in southern France demonstrated a similar pattern of declining P levels in the presence of lower organic acid concentrations, which significantly reduced P levels at 20 µmol/L of added citrate (Duputel et al. 2013b). An explanation for this decline in P availability could be the high amount of sorption of organic acids (particularly oxalate) at these lower concentrations compared to the higher concentrations (Ström et al. 2001). Another possibility could be “Ca-aided co-adsorption,” where low amounts of organic acids might increase the adsorption of Ca^2+^ ions, sequentially increasing P adsorption (Duputel et al. 2013a, b). Contrastingly, we observed the opposite trend with organic P levels (Supplementary Appendix 4, Figure A4-1), where in a few cases, especially at the igneous alluvium site, there is some evidence that organic P is released at a median concentration of 50 µmol/L across all three acids (*p* ≤ 0.0005), though the amounts were small compared to overall water-extractable P pool sizes. The highest organic P extraction occurred with malate, followed by oxalate and citrate. Additionally, the igneous alluvium site is the most acidic compared to other sites. Previous studies that suggest that organic acids promote organic P release were conducted in acidic forest soils of China and Australia (Wei et al. 2010; Hou et al. 2018; Zhu et al. 2022), which may thus be the key difference with our alkaline dryland soils.

## Conclusion

Our study contributes to an improved understanding of the interaction between soil P and organic acids in dryland soils. The results suggest that PO_4_^3−^-release thresholds via organic acids (citrate and oxalate) are broadly consistent across different site characteristics and microhabitats (under plant canopies vs. interspaces), indicating a generalized response in the tested dryland soils. The relatively high concentrations (on the order of 1000 µmol/L) are needed for organic acids to positively affect inorganic P dissolution from Ca-P complexes, while organic P release in these soils was not substantial. The in situ organic acid concentrations add important context to this conclusion, indicating that the bulk soil concentration of organic acids was not effective/critical enough to improve P mobilization. Still, there could be localized hotspots, likely in the rhizosphere, that may approach or exceed these higher organic acid concentrations, making this mechanism still potentially viable, although spatially and temporally limited. Estimates of true rhizosphere concentrations are highly variable and warrant further research. Given that the threshold is reached, our results suggest that oxalate, as opposed to citrate and malate, would be most effective in mobilizing P, likely via calcium oxalate formation and precipitation, which binds calcium and releases phosphate from Ca-P complexes. Overall, these results suggest that dryland soil organisms either rely on localized concentration hotspots to utilize organic acids for PO_4_^3−^ mobilization or employ a different P acquisition strategy.

## Supplementary Information

Below is the link to the electronic supplementary material.

Supplementary file1 (DOCX 215 KB)

## Data Availability

The datasets generated during and/or analyzed during the current study are available in the HydroShare repository (Kukreja and Darrouzet-Nardi 2025, 10.4211/hs.54e6ba56bcd04e08b5fbc1e8cc96b27d).
