# Supplementary material for: Organic acid concentration thresholds to mobilize phosphorus (P) in dryland soils
Source: Biogeochemistry. 2025 Dec 20;169(1):4. doi: 10.1007/s10533-025-01298-5 (PMC12804238; doi:10.1007/s10533-025-01298-5)
Supplement: Supplementary file 1 — Supplementary file1 (DOCX 215 KB) [file 10533_2025_1298_MOESM1_ESM.docx]

**Supplementary Information for**

Organic acid concentration thresholds to mobilize phosphorus (P) in dryland soils

Kalpana Kukreja^1*^, Elizabeth Noriega Landa^2^, Wen-Yee Lee^2^, Mark A. Engle^3^, Anthony Darrouzet-Nardi^1^

1. Department of Biological Sciences, University of Texas at El Paso, El Paso, Texas, USA
2. Department of Chemistry, University of Texas at El Paso, El Paso, Texas, USA
3. Department of Earth, Environmental and Resource Sciences, University of Texas at El Paso, El Paso, Texas, USA

*Corresponding author – [kkukreja@miners.utep.edu](mailto:kkukreja@miners.utep.edu)

ORCID iD - [0000-0002-8995-1288](https://orcid.org/0000-0002-8995-1288)

This supplementary information file includes additional methods, data analysis, extended results, and discussions cited in the main manuscript. Phosphorus concentrations in soil extracts were measured using two methods: the malachite green colorimetric assay and inductively coupled plasma–optical emission (ICP-OES). A comparison of these methods revealed a 92% correlation (Figure A1-1). A brief result and discussion are provided to interpret this correlation, along with the reference list in Appendix 1. The detailed description of gas chromatography-mass spectrometry (GC-MS) parameters is provided in Appendix 2 (Table A2-1). All fixed effects interactions were tested using a linear mixed-effects model ANOVA and are reported in Appendix 3 (Table A3-1). We further examined the significant variables for their specific contrasts to supplement our ANOVA results, also shown in Appendix 3 (Tables A3-2 and A3-3). Responses of organic P across tested organic acids and site characteristics are presented in Appendix 4 (Figure A4-1). Appendix 4. Additional discussions on site differences are included in Appendix 5.

**Appendix 1**

**Method comparison (Malachite green colorimetric assay vs. ICP-OES)**

We measured phosphate concentrations using ICP-OES (Figure 5), and the results were comparable to those of the microplate method, showing a correlation of *r*^2^ = 0.92 between the two methods (Figure A1-1).


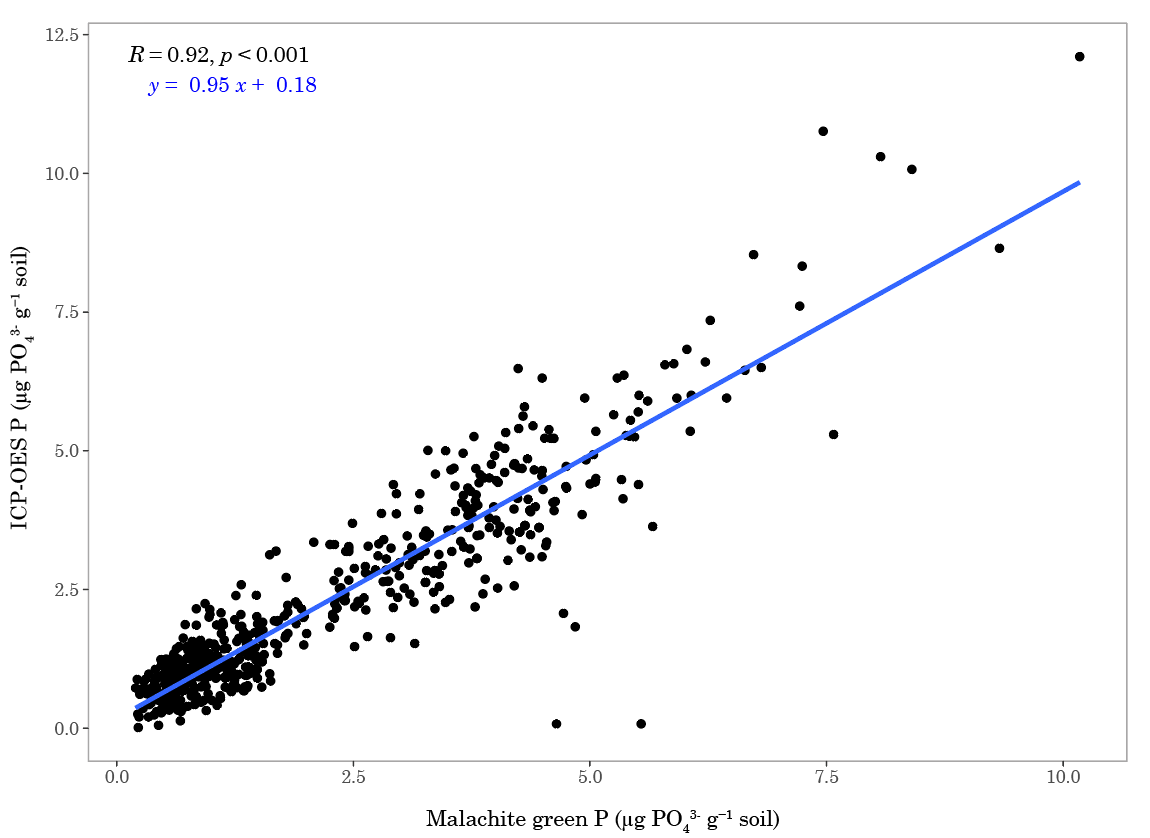


Figure A1-1. Correlation between ICP-OES P and Malachite green P.

To measure P, our data indicates a strong correlation between the two methods (Malachite green and ICP-OES), suggesting the reliability of both methods for P measurements. A lab experiment demonstrated that ICP-OES has the potential to determine total dissolved phosphorus, while malachite green only measures inorganic phosphorus (orthophosphate) (Shwiekh et al. 2013). The ratio of malachite green to ICP-OES P is 1:1.25; the slightly higher ICP-OES value could be either due to suspended colloidal P and soluble complexes of P with Ca or could be organic P and polyphosphates (Shwiekh et al. 2013). Additionally, organic acids as a carbon source could enhance microbial activity, which further help to release organic P (Wang et al. 2015).

**References**

Hartley A, Barger N, Okin G (2007) Dryland Ecosysytems. In: Nutrient Cycling in Terrestrial Ecosystems. Springer Science & Business Media, pp 271–307

Shwiekh R, Kratz S, Schick J, et al (2013) Determination of inorganic and organic P dissolved in water and Olsen extracts by inductively coupled plasma optical emission spectroscopy (ICP-OES) and colorimetry/Bestimmung von anorganischem und organischem P im Wasser- und Olsenextrakt mittels optischer Emissionsspektroskopie (ICP-OES) und Kolorimetrie. Landbauforschung 63:303–307. https://doi.org/10.3220/LBF_2013_303-306

Wang Y, Chen X, Whalen JK, et al (2015) Kinetics of inorganic and organic phosphorus release influenced by low molecular weight organic acids in calcareous, neutral and acidic soils. Journal of Plant Nutrition and Soil Science 178:555–566. https://doi.org/10.1002/jpln.201500047

**Appendix 2**

*Table A2-1. GCMS Method Parameters.*

| TD-CGMS Parameters | |
| --- | --- |
| Gas Flow (mL/min) | 1.2 |
| Column | HP-5ms Ultra Inert capillary |
| Column dimension | 30 m x 0.25 µm x 0.25 µm |
| Desorption mode | Splitless |
| Acquisition mode | SIM/Scan |
| Scan Range (m/z) | 14-290 |
| SIM ions | 15, 45, 59, 71, 101,  103, 143, 272 |
| TD 3.5+ | |
| Initial Temp (°C) | 50 |
| Hold time (min) | 0.5 |
| 1st Rate (°C/min) | 100 |
| 1st Temp (°C) | 280 |
| 1st Hold Time (min) | 5 |
| CIS4 | |
| Initial Temp (°C) | -40 |
| 1st Rate (°C/min) | 12 |
| 1st Temp (°C) | 300 |
| 1st Hold Time (min) | 5 |
| Oven | |
| Initial Temp (°C) | 50 |
| Hold time (min) | 4 |
| 1st Rate (°C/min) | 10 |
| 1st Temp (°C) | 70 |
| 2nd Rate (°C/min) | 10 |
| 2nd Temp (°C) | 170 |
| 3rd Rate (°C/min) | 25 |
| 3rd Temp (°C) | 250 |
| Final Hold time (min) | 10 |

**Appendix 3**

Table A3-1. ANOVA table for the mixed-effects model used to determine the significance of fixed effects and their interactions.

|  | numDF | denDF | F-value | p-value |
| --- | --- | --- | --- | --- |
| 1. **Phosphorus** | | | | |
| (Intercept) | 1 | 480 | 728.8090 | <.0001 |
| **treatment** | 6 | 480 | 483.5977 | <.0001 |
| **organic acid** | 2 | 480 | 167.8276 | <.0001 |
| microhabitat | 1 | 24 | 0.1261 | 0.7256 |
| **landform** | 2 | 24 | 142.1902 | <.0001 |
| **treatment * organic acid** | 12 | 480 | 116.0611 | <.0001 |
| microhabitat * landform | 2 | 24 | 0.3092 | 0.7369 |
| treatment * organic acid * microhabitat | 12 | 480 | 0.3840 | 0.9691 |
| **treatment * organic acid * landform** | 24 | 480 | 3.5915 | <.0001 |
| treatment * microhabitat * landform | 12 | 480 | 0.9658 | 0.4807 |
| organic acid * microhabitat * landform | 4 | 480 | 1.1466 | 0.3339 |
| treatment * organic acid * microhabitat * landform | 24 | 480 | 0.7105 | 0.8428 |
| 1. **Calcium** | | | | |
| (Intercept) | 1 | 480 | 5109.512 | <.0001 |
| **treatment** | 6 | 480 | 6300.134 | <.0001 |
| **organic acid** | 2 | 480 | 3721.426 | <.0001 |
| microhabitat | 1 | 24 | 1.717 | 0.2025 |
| **landform** | 2 | 24 | 369.943 | <.0001 |
| **treatment * organic acid** | 12 | 480 | 3494.846 | <.0001 |
| microhabitat * landform | 2 | 24 | 0.619 | 0.5466 |
| treatment * organic acid * microhabitat | 12 | 480 | 1.140 | 0.3253 |
| **treatment * organic acid * landform** | 24 | 480 | 100.682 | <.0001 |
| **treatment * microhabitat * landform** | 12 | 480 | 5.306 | <.0001 |
| organic acid * microhabitat * landform | 4 | 480 | 0.902 | 0.4625 |
| treatment * organic acid * microhabitat * landform | 24 | 480 | 1.656 | 0.0270 |

Table A3-2. Post-hoc emmeans contrasts of the response variable phosphorus from the linear mixed effect model, comparing treatment to the control within each organic acid. p values are adjusted using Dunnettx’s method. Significant differences correspond to P < 0.0001 ***, < 0.001 **, < 0.05 *

| 1. **Citrate** | | | | | |
| --- | --- | --- | --- | --- | --- |
| Contrast | Estimate | SE | df | t.ratio | p.value |
| 10 µmol/L - Control | -0.2781 | 0.0865 | 480 | -3.216 | 0.0077* |
| 20 µmol/L - Control | -0.1932 | 0.0865 | 480 | -2.235 | 0.1205 |
| 50 µmol/L - Control | -0.3326 | 0.0865 | 480 | -3.847 | 0.0008** |
| 100 µmol/L - Control | -0.2232 | 0.0865 | 480 | -2.581 | 0.0512 |
| 1000 µmol/L - Control | 0.6830 | 0.0865 | 480 | 7.898 | <.0001*** |
| 10000 µmol/L - Control | 1.7902 | 0.0865 | 480 | 20.702 | <.0001*** |
| 1. **Malate** | | | | | |
| Contrast | Estimate | SE | df | t.ratio | p.value |
| 10 µmol/L - Control | -0.0476 | 0.0865 | 480 | -0.550 | 0.9545 |
| 20 µmol/L - Control | -0.1778 | 0.0865 | 480 | -2.056 | 0.1775 |
| 50 µmol/L - Control | -0.3621 | 0.0865 | 480 | -4.187 | 0.0002** |
| 100 µmol/L - Control | -0.3386 | 0.0865 | 480 | -3.916 | 0.0006** |
| 1000 µmol/L - Control | -0.0447 | 0.0865 | 480 | -0.517 | 0.9618 |
| 10000 µmol/L - Control | 0.1200 | 0.0865 | 480 | 1.388 | 0.5333 |
| 1. **Oxalate** | | | | | |
| Contrast | Estimate | SE | df | t.ratio | p.value |
| 10 µmol/L - Control | -0.1942 | 0.0865 | 480 | -2.246 | 0.1175 |
| 20 µmol/L - Control | -0.2762 | 0.0865 | 480 | -3.194 | 0.0083* |
| 50 µmol/L - Control | -0.3741 | 0.0865 | 480 | -4.326 | 0.0001** |
| 100 µmol/L - Control | -0.2357 | 0.0865 | 480 | -2.726 | 0.0345* |
| 1000 µmol/L - Control | 0.8377 | 0.0865 | 480 | 9.687 | <.0001*** |
| 10000 µmol/L - Control | 3.5767 | 0.0865 | 480 | 41.361 | <.0001*** |

Table A3-3. Post-hoc emmeans contrasts of the response variable calcium from the linear mixed effect model, comparing treatments to the control within each organic acid. p values are adjusted using Dunnettx’s method. Significant differences correspond to P < 0.0001 ***, < 0.001 **, < 0.05 *

| 1. **Citrate** | | | | | |
| --- | --- | --- | --- | --- | --- |
| Contrast | Estimate | SE | df | t.ratio | p.value |
| 10 µmol/L - Control | -6.6412 | 3.99 | 480 | -1.666 | 0.3618 |
| 20 µmol/L - Control | -5.1977 | 3.99 | 480 | -1.304 | 0.5881 |
| 50 µmol/L - Control | -0.0533 | 3.99 | 480 | -0.013 | 1.0000 |
| 100 µmol/L - Control | 12.7144 | 3.99 | 480 | 3.189 | 0.0084 |
| 1000 µmol/L - Control | 114.7985 | 3.99 | 480 | 28.796 | <.0001*** |
| 10000 µmol/L - Control | 798.1902 | 3.99 | 480 | 200.215 | <.0001*** |
| 1. **Malate** | | | | | |
| Contrast | Estimate | SE | df | t.ratio | p.value |
| 10 µmol/L - Control | 19.14 | 3.99 | 480 | 4.802 | <.0001*** |
| 20 µmol/L - Control | 3.12 | 3.99 | 480 | 0.784 | 0.8794 |
| 50 µmol/L - Control | 105.46 | 3.99 | 480 | 26.452 | <.0001*** |
| 100 µmol/L - Control | 78.52 | 3.99 | 480 | 19.694 | <.0001*** |
| 1000 µmol/L - Control | 92.91 | 3.99 | 480 | 23.306 | <.0001*** |
| 10000 µmol/L - Control | 358.16 | 3.99 | 480 | 89.839 | <.0001*** |
| 1. **Oxalate** | | | | | |
| Contrast | Estimate | SE | df | t.ratio | p.value |
| 10 µmol/L - Control | -4.54 | 3.99 | 480 | -1.139 | 0.6932 |
| 20 µmol/L - Control | 46.54 | 3.99 | 480 | 11.675 | <.0001*** |
| 50 µmol/L - Control | 41.71 | 3.99 | 480 | 10.462 | <.0001*** |
| 100 µmol/L - Control | 33.89 | 3.99 | 480 | 8.502 | <.0001*** |
| 1000 µmol/L - Control | -24.66 | 3.99 | 480 | -6.185 | <.0001*** |
| 10000 µmol/L - Control | -63.50 | 3.99 | 480 | -15.929 | <.0001*** |

**Appendix 4**


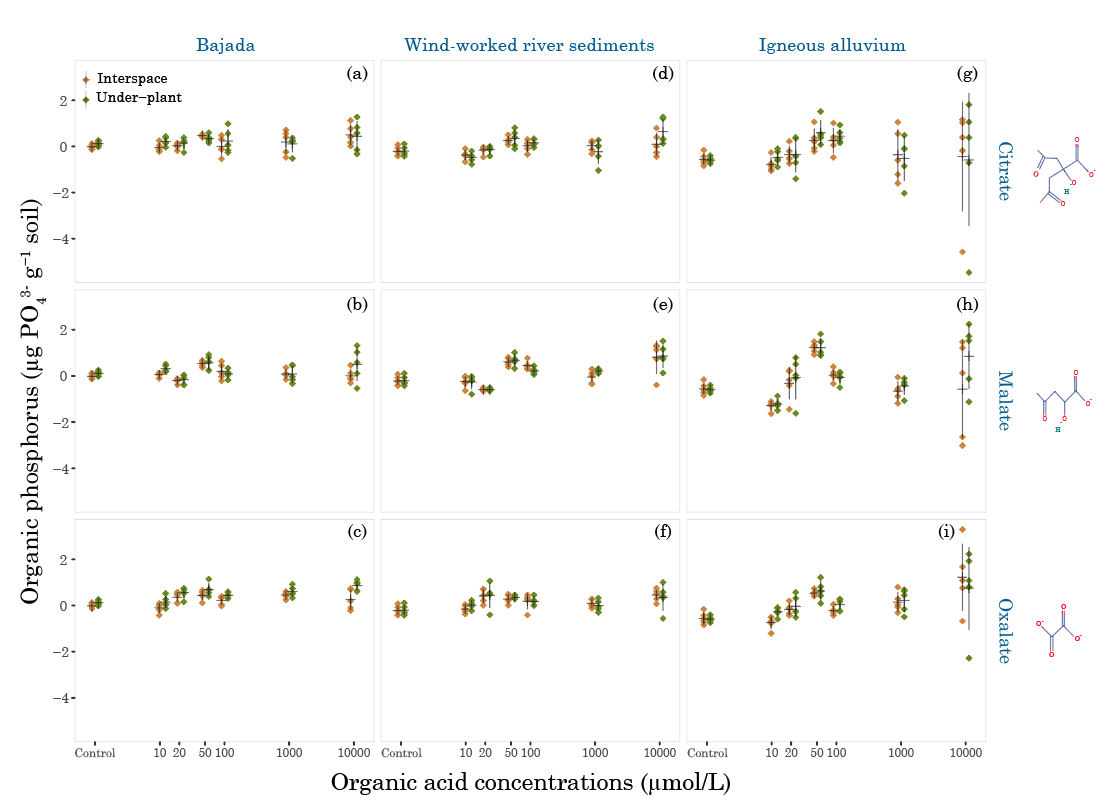


Figure A4-1. Organic phosphorus as a response to varying organic acids, concentrations, landforms, and microhabitats. The X-axis is logarithmic, and each PO_4_^3-^ value (mean ± SE; denoted by a black cross) is the average of five sampling locations at each site. Note: The difference between total dissolved phosphorus (measured by ICP-OES) and inorganic phosphorus (determined by the malachite green colorimetric assay) is regarded as organic P.

**Appendix 5**

**Site differences**

We observed notable differences between the sites with different parent materials and landforms. The igneous site, where we detected higher total P in bulk soils (via XRF), also had higher PO43- levels, while the bajada site showed lower total P and PO43-; the parent material is a possible link. However, we have only three sites in this study, so more would be needed to establish a relationship. Additionally, our data showed an opposite trend for total Ca in bulk soil (5.9%, Table 3), water-extractable Ca2+, and soil inorganic carbon (SIC) concentrations (1.08%, Table 3), which were higher at the bajada site compared to the levels of PO43- and total P across the studied sites. High Ca2+ and HCO3– concentrations are known to limit P availability by reducing the solubility of carbonate and Ca-P complexes, or remaining undissolved (Hartley et al. 2007).
